# The effect of need supportive text messages on motivation and physical activity behaviour

**DOI:** 10.1007/s10865-016-9722-1

**Published:** 2016-02-26

**Authors:** Florence-Emilie Kinnafick, Cecilie Thøgersen-Ntoumani, Joan Duda

**Affiliations:** Institute of Health and Well-being, University of Northampton, Northampton, NN2 7AL UK; School of Psychology and Speech Pathology, Curtin University, Bentley, WA 6102 Australia; School of Sport, Exercise and Rehabilitation Sciences, University of Birmingham, Birmingham, B15 2TT UK

**Keywords:** SMS, Need support, Exercise class, Intervention, Self-determination theory

## Abstract

Few short messaging service (SMS) studies to support behaviour change have used a theoretical underpinning. Using a self-determination theory perspective, we explored the effects of need supportive (NS) SMS on physical activity in 65 (BMI = 24.06 kg/m^2^, *SD* = 5.49; *M* = 25.76 years, *SD* = 10.23) insufficiently active individuals embarking on an existing exercise programme. For 10 weeks participants were randomised to an intervention group (NS) or control group (neutral). SMS were sent twice weekly, randomly, via an online SMS service. Mixed design ANCOVA and MANCOVA analyses of measures taken at baseline, mid and post intervention revealed increased levels of perceived autonomy support and psychological need satisfaction in the intervention group post intervention. Both groups reported increases in intrinsic motivation from pre to post intervention. Moderate intensity physical activity was greater in the intervention than the control group at 4-month post intervention with control group returning to baseline levels. Findings provide preliminary causal evidence to support the use of NS SMS to optimise physical activity behaviour change in individuals who are insufficiently active.

**Electronic supplementary material:**

The online version of this article (doi:10.1007/s10865-016-9722-1) contains supplementary material, which is available to authorized users.

## Introduction

Despite a wealth of evidence showing that physical inactivity contributes to overweight, obesity (Department of Health, 2011) and chronic non-communicable diseases (Lee et al., 2012), over two-thirds of the population in the UK (60 % of adult males and 75 % of adult females) do not meet the recommended levels of physical activity to improve or maintain health. Current public health guidelines encourage individuals between the ages of 18–64 years old to accumulate 150 min (2.5 h) of moderate intensity activity in bouts of 10 min or more *or* 75 min of vigorous intensity exercise per week (Department of Health, 2011). Physical activity interventions aimed at increasing levels of physical activity in physically inactive populations have shown significant benefits to both physical (Haerens et al., 2006) and psychological well-being (Netz et al., 2005). However, adherence to a physically active lifestyle is still a concern as physical activity levels typically decline following the end of an intervention (Fuchs et al., 2011). There is an urgent need to identify effective interventions to optimise long term physical activity behaviour.

More recently, technological devices have been introduced to support health behaviour changes. Text messaging or short message service (SMS) operates on essentially all mobile phones. With over seven billion mobile-cellular subscriptions, and approximately 87 % of the global population owning a mobile phone (ITU, 2015), a SMS can be a powerful health promotion tool to reach a large portion of the population instantly, with little expense, and without requiring extensive technological expertise (Krishna et al., 2009). SMS have been used with some success as a tool in conjunction with other intervention components, to facilitate smoking cessation (Berkman et al., 2011), change sexual behaviour practices (Gold et al., 2011), facilitate weight loss (Shapiro et al., 2012) and to optimise physical activity behaviour change (Kim & Glanz, 2013). Given the immature nature of the field and the focus on interventions with clinical populations (e.g., diabetes management: Newton et al., 2009), further research is needed for preventive health care. Extant research has also lacked general scientific rigour (Cole-Lewis & Kershaw, 2010). For example, most studies have not isolated the effect of text message technology (e.g., Newton et al., 2009) but have used text messages in conjunction with other intervention components and few have examined the longer-term effects, including follow up periods, of text messages on health behaviour (e.g., Hurling et al., 2007; Kim & Glanz, 2013). Fjeldsoe et al. (2010)’s text message based intervention (MobileMums) resulted in increases in moderate and vigorous intensity physical activity although the participants reported high use of other intervention components (e.g., social support, a goal setting fridge magnet). Cole-Lewis and Kershaw (2010) emphasised that text messages should not be a standalone model for behaviour change. Equally, however, it is important to understand the extent that the text messages themselves, can increase the motivation of an individual whilst attempting to minimise the influence of confounding variables (e.g., significant other, paper diaries, pedometers, emails). Shapiro et al. (2012) found that adherence to the text messaging was associated with improvement in weight-related behaviours and weight outcomes at 6 and 12 months. Shapiro et al. (2012), however, like Hurling et al. (2007) and Kim and Glanz (2013) used a financial incentive and did not include any follow up assessment.

Developing and designing appropriate messages within the limited character space available (160 characters) is a challenge and has been the focus of some, albeit limited, research (Hingle et al., 2012; Redfern et al., 2012). Limited information on specific process measures has been reported on message development and content within text message interventions (Cole-Lewis & Kershaw, 2010) making the assessment of delivery mechanisms difficult (Whittaker et al., 2009).

Research has shown that messaging interventions grounded in behavioural theory are more likely to be successful in changing the targeted behaviour, allowing for better understanding of the mechanisms of change (Van’t Riet et al., 2010). Few studies involving text messages have been underpinned by theory (Cole-Lewis & Kershaw, 2010; Shapiro et al., 2012). While some researchers have attempted to incorporate constructs of multiple behaviour change theories, they have not been explicit in the description of the theoretical constructs that are being targeted (Fjeldsoe et al., 2009).

Self-determination Theory (SDT; Deci & Ryan, 1985, 2000) is a macro-motivational theory of behaviour change applied successfully to a range of life settings, including health and physical activity (Ng et al., 2012; Teixeira et al., 2012). SDT theorists posit that an individual will possess more or less self-determined motivation to engage in a particular behaviour (e.g., physical activity). It is proposed that the quality of motivation lies on a continuum which distinguishes types of behavioural regulation varying in the extent to which they are autonomous; autonomous motivation (intrinsic motivation, integrated regulation, identified regulation), controlled motivation (introjected regulation, external regulation) and amotivation (Ryan & Deci, 2000).

Amotivation represents the lack of either intrinsic or extrinsic motivation. External regulation is evident when an individual engages in a behaviour because of external pressures; to satisfy others or for a financial incentive. This type of motivation has consistently been shown to be a negative predictor of adherence to physical activity (Teixeira et al., 2012). An individual who displays high levels of introjected regulation engages in a behaviour out of feelings of internal pressure generally posited to be associated with more maladaptive outcomes such as negative affect, feelings of guilt and lowered self-esteem (Deci & Ryan, 2000). However, Thøgersen-Ntoumani and Ntoumanis (2006) showed that both introjected and identified regulation can be associated with positive outcomes (e.g., intentions). Identified regulation is an autonomous form of extrinsic motivation whereby the individual recognises the benefits and value of the behaviour (i.e., health benefits as a result of a physically active lifestyle). Integrated regulation is considered the most self-determined extrinsic regulation (Markland & Tobin, 2004). An individual showing high levels of integrated regulation will engage in a behaviour coherent with other values and aspirations (Deci & Ryan, 2000). Finally, intrinsic motivation is evident when an individual participates in a behaviour because of an inherent interest in the activity and is associated with positive and sustained behavioural outcomes in the health domain (Ng et al., 2012).

SDT theorists suggest that all individuals have three key psychological needs (the need for autonomy, competence and relatedness) which must be satisfied to optimise the quality of motivation for behavioural adoption and maintenance within any particular context (Deci & Ryan, 2000). The need for autonomy refers to a need for feelings of volition and free will; the sense that the individual is in control of his or her own behaviours and feels empowered. Individuals also need to feel competent or effective in carrying out behaviours and handling situational demands. Finally, people have a need to feel related, connected to, and accepted by significant others in the given context.

According to SDT, the social contextual environment can facilitate the internalisation process whereby an individual begins to actively endorse the utility of performing a particular behaviour and this, in turn, is shown to predict adaptive outcomes (e.g., behavioural engagement and maintenance; Fortier et al., 2012). As Teixeira et al. (2012) explain, need fulfilment and optimising the quality of motivation is associated with whether a significant other who plays an instrumental role in shaping an individual’s experience within a particular domain (e.g., exercise instructors or health practitioners), supports the need for autonomy, relatedness and competence and thus promotes the intrinsic interest of the activity. Examples of need supportive behaviour include: Providing choice, a meaningful rationale, minimising pressure, acknowledging the perspective of the participant (autonomy), acknowledging negative feelings associated with the behaviour, demonstrating unconditional regard, provides understanding and care (relatedness) and for competence, providing advice on resisting and overcoming barriers and, providing optimally challenging tasks (Rouse et al., 2011; Williams et al., 2006).

Intervention research in the physical activity domain has provided support for these motivational processes (Teixeira et al., 2012). Moustaka et al. (2012) and Edmunds et al. (2008) focused on facilitation of autonomy need satisfaction when they tested an autonomy supportive teaching style within the context of an exercise class (8 and 10 weeks, respectively). The intervention group in both studies reported increases in autonomous motivation. The participants in the intervention group in the study by Moustaka et al. (2012) experienced increases in autonomy and competence need satisfaction while relatedness and competence need satisfaction were the psychological needs key to predicting positive outcomes in the intervention group participants in the study by Edmunds et al. (2008). It was not possible, in either study, to determine whether other influences beyond the instructor provided autonomy support and their independent roles in achieving the desired outcomes. Further research is therefore needed to explore who, or indeed what, can provide need support and the unique impact of each source on the individual’s initial motivation and behavior.

Current research has mostly focused on creating an autonomy supportive environment through a figure of authority (i.e., exercise professional) (Moustaka et al., 2012). However, Kinnafick et al. (2014) found that satisfaction of the needs for autonomy and relatedness could be supported via different sources (a walk leader and a walking programme as a whole). To our knowledge, there has only been one other published article on the proposal of text messages grounded in SDT, aimed at increasing physical activity behaviour (Thompson et al., 2014). This research was completed in an adolescent population, and used pedometers and self-selected step goals in conjunction with the messages. No SDT grounded research, using an adult population, has previously investigated the possibility that a text message from a mobile phone, can provide a source of need support.

### Aims and hypotheses

In light of the existing research and using a randomised controlled design, our overarching aim was to investigate the unique effects of text messages based on principles of SDT, compared to neutral text messages, in the promotion of physical activity behaviour in a physically inactive population beginning a series of exercise classes. We aimed to investigate these effects whilst controlling for a variable (participants perception of autonomy support from the exercise class instructor) that has previously shown to affect outcomes (Moustaka et al., 2012). We proposed that those in the intervention group will report greater levels of perceived autonomy support (H1) need satisfaction (H2) and experience greater self-determined motivation in comparison to the control group (H3). It is also hypothesised that those receiving need supportive text messages will experience greater increases in self-reported physical activity (H4: including at a 4-month follow up), compared to the control group (neutral text messages).

## Methods

### Pilot and message development

We conducted a pilot study to test the content of the text messages prior to the main study. This was to determine if the messages were perceived as either need supportive or neutral. A list of neutral (30 messages) and need supportive messages (70 messages: based on environmental support for autonomy, competence and relatedness), written by the first author, were randomly mixed into one questionnaire and given to five researchers with expertise in self-determination theory (doctoral researchers and academic staff). The strategies included in the messages according to each construct can be seen in Table 1. We aimed to include as many of the strategies into each single text as possible. In doing so we did not separate the messages by construct (autonomy, competence and relatedness). At baseline, prior to random allocation, all participants rated their motivation to exercise via the Exercise Motivation Inventory (EMI-2; Markland & Hardy, 1993). Data collected from the EMI-2 helped us to frame the messages in a need supportive way specifically targeting the behavioural strategy of acknowledging individual perspective and allowing us to accurately promote the intrinsic interest of the exercise. The number of messages created for the pilot was calculated to ensure that participants would receive unique messages according to their 5 most highly rated motives.

**Table 1:** Dimensions of SDT included in the need supportive texts in relation to each basic psychological need

Autonomy	Providing information as a meaningful rationale
	Enhance perceptions of value of activity
	Provision of choice and variety
	Facilitating enjoyment
Relatedness	Social support: trusting and feeling connected to others
	Portray respect, understanding and care
	Acknowledge negative feelings associated regarding the activity
Competence	Supporting confidence by providing information and relevant feedback
	Help setting challenging but realistic goals
	Advice on resisting and overcoming barriers
	Focus on intrinsic goals i.e., health, personal growth

The field experts rated the text messages based on how need supportive they perceived the messages to be on a scale ranging from 1 (not at all need supportive) to 7 (highly need supportive). We also encouraged them to provide further comments and feedback. Text messages (examples can be seen in Table 2) were modified accordingly based on the results and feedback of the pilot study.

**Table 2:** Examples of autonomy supportive text messages and neutral messages

Autonomy supportive text messages
(1) Enjoyment: *Hi…! We understand that you may not always feel like going to your exercise class; if* *you are busy or the weather is bad. Perhaps try and think about the elements you enjoy, whether it’s your favourite exercise or instructor?*
(2) Affiliation: *Hi…! You indicated that the social aspect of your physical activity is important to you. Exercising with friends can increase the positives of physical activity further! Who said gossiping was a bad thing?!*
(3) Ill-Health Avoidance: *Hi…! Osteoporosis, a common disease as you age can be offset by physical activity: no matter your age! It is possible for you to accrue the benefits by remaining physically active!*
Neutral text messages
(1) *Physical activity is defined as “any force exerted by skeletal muscle that results in energy expenditure above resting level”*
(2) *The British population spends around £110 billion on healthcare per year which is equal to 8.5* *% of all income*
(3) *The Government has set a target in England and Wales for 70* *% of the population (in Wales, people up to the age of 65) to be ‘reasonably active’ by 2020*

### Participants

**Fig. 1:**
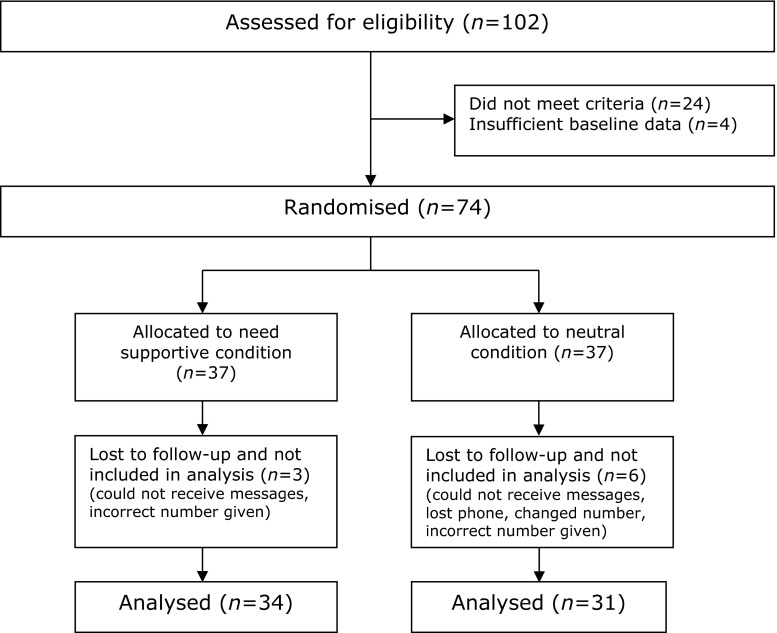
CONSORT flowchart showing participants progress through study

Following approval from an ethics board within a large UK University, a total of 102 participants (staff and students of the University) volunteered to participate in the study and provided informed consent. Participants were included if they were physically inactive according to the recommendation for physical activity set for adults by the Department of Health (2011), owned a mobile phone and were participating or intended to participate in their first week of exercise classes following a physically inactive period of at least 2 years. Due to the constraints of the academic term, the recruitment phase was restricted to a 1-week period. Over that 1-week period, the first author attended a total of 25 exercise classes, out of a possible 32, that were part of the existing ‘Active Lifestyles’ programme at the university sport centre to recruit possible participants. The classes consisted of a range of activities including aerobic, toning or dance elements. A CONSORT diagram showing participant progress through the study can be seen in Fig. 1. We included a total of 65 physically inactive participants in the analysis of the 10 week intervention (*n* = 61 females, 4 male; BMI = 24.06 kg/m^2^, *SD* = 5.49; age range 18–66 years, *M* = 25.76, *SD* = 10.23).

### Intervention

Using an online random number service (www.random.org) we randomly allocated participants to one of two groups, the intervention group (*n* = 34; ‘need supportive SMS’) or the control group (*n* = 31; ‘neutral SMS’). We gave no instructions on how many or which exercise classes to attend. Each participant received two text messages per week to their personal mobile phones for a total of 10 weeks. Two text messages were sent per week to reflect the expected frequency of the target behaviour (Fjeldsoe et al., 2009) in order to meet the recommended guidelines for vigorous physical activity (75 min/week). Two classes of 60 min would meet those recommendations. The length of the intervention was determined by the duration of the structured group exercise programme which took place only during the University spring term.

The intervention group received need supportive framed messages. To keep in line with principles of SDT and need support, the participants who reported extrinsically oriented motives (i.e., weight management) were sent messages focusing on the personal value that the motive could hold for the participant. An example message for a participant who specified losing weight as a priority motive was, “Losing weight as a result of physical activity can have a positive influence on your mental well-being by increasing your self-confidence!” Participants in the intervention group received text messages targeted to their five most important (i.e., highest scoring) motives. The control group received neutrally framed text messages. Each participant received a different message with each text. One-way directional text messages were sent at random times (scheduled ahead of the intervention by www.random.org) between 9 a.m. and 6 p.m. Monday to Friday via an online text service (www.textanywhere.com). Participants were not required to reply but did have the option should they want to comment or ask a question. The university exercise class system works on a credit basis. This approach means that class attendees buy a pre-specified number of credits and choose the classes they wish to attend. Therefore the text messages were sent at random times throughout the week.

We asked participants to complete a questionnaire and return it via post (they were given a pre-paid self-addressed envelope) or via the university’s internal postal system. Questionnaires were completed at baseline (week 0), at mid-intervention (week 5), and post intervention (week 11). Self-report physical activity was also measured at 4 months following the end of the intervention (6 months from starting the intervention). The follow up period was again determined by the academic term.

### Measures

#### Anthropometric measures

We recorded height, measured with a Stadiometer, and weight, measured with an Omron BF508 Body Composition Scales (Omron, UK), at the point of recruitment in order to calculate participants’ Body Mass Index (BMI).

#### Motivation to exercise

We measured all participants’ specific motives for engaging in exercise at baseline using the EMI-2 (Markland & Hardy, 1993). The EMI-2 classifies motives into 14 categories (e.g., stress management, enjoyment, social recognition). Some motives are intrinsically-oriented (i.e., enjoyment) while others are extrinsically oriented (i.e., weight management).

#### Autonomy support

We assessed perceived autonomy support mid and post intervention using the validated 15 item Health-Care Climate Questionnaire (HCCQ: Williams et al., 1996) adapted to an exercise setting. We measured the degree of perceived autonomy support provided by the exercise class instructor (e.g., *I feel the instructor has provided me with choices and options*) and the text messages (e.g., *The text messages convey confidence in my ability to make changes*) separately. Participants responded to the autonomy support items on a 7-point likert scale (1 = *Strongly disagree*; 7 = *strongly agree*). Cronbach alpha values from the current study ranged between .75 and .96 across both groups.

#### Psychological need satisfaction

We measured participants’ perceptions of psychological need satisfaction (Baard et al., 2004) derived from the text messages (e.g., Autonomy: *The text messages take my feelings regarding exercising into consideration*, Competence: *The text messages help me feel a sense of accomplishment from exercising*, Relatedness: *The text messages demonstrate caring about me as a person*) using a smaller, 9 item version of the 21-item Basic Need Satisfaction at Work Scale (Deci et al., 2001), namely 3 items per sub-scale. The smaller scale was used as some items in the longer scale were not deemed to be relevant in relation to psychological need satisfaction from the text messages (e.g., “I get along with the people in my exercise class”). Participants responded to the items on a 7-point likert scale ranging from 1 (*strongly disagree*) to 7 (*strongly agree*) at mid intervention (week 5) and post intervention (week 11). The 9 item scale has shown internal reliability (Cronbach alpha scores above .90) in previous research (La Guardia et al., 2000). Cronbach alpha values from the current study ranged from .71 to .93 in the intervention group and the control group.

#### Behavioural regulations to exercise

We measured participants’ quality of motivation for engaging in exercise at baseline and post intervention using the Behavioural Regulations to Exercise Questionnaire (BREQ-2; Markland & Tobin, 2004). The scale consists of 19 items and participants are asked to respond to the questions in terms of reasons for engaging in exercise. They were rated on a 5-point likert scale ranging from 0 (*not true for me*) to 4 (*very true for me*). The five subscales within the questionnaire represent intrinsic motivation (e.g., *I take part in the exercise class because it is fun*), identified regulation (e.g., *I take part in the exercise class because I value the benefits of exercising*), introjected regulation (e.g., *I take part in the exercise class because I feel guilty when I don’t exercise*), external regulation (e.g., *I take part in the exercise class because others say I should*), and amotivation (e.g., *I take part in the exercise class but I don’t see why I should have to exercise*). Cronbach alpha scores in the current study across both groups ranged from .73 to .92.

#### Physical activity behaviour

We used the 7-day physical activity recall (7 Day-PAR; Blair et al., 1985) to determine the duration and intensity of physical activities in the past week. At the point of recruitment we asked participants (via interview with the first author) to recall time spent engaging in different types of physical activity throughout the past 7 days (or a typical week of the last month, if the previous week was atypical). We administered this measure at baseline, before the participants were randomly allocated to a group (for a baseline measure and eligibility for the study), post intervention and 4 month following the end of the intervention. At post intervention and at follow up, the first author carried out the interview over the phone. In order to minimise bias at these time points the first author was blinded to which group each participant was allocated and identification numbers were matched up to the relevant group following the collection of this data. We gave participants examples of typical activities for moderate and vigorous intensities and rated each of them in minutes of time spent that week. We summed values from ‘moderate’ and ‘vigorous’ categories of intensity separately to give scores (in minutes) of moderate intensity and vigorous intensity physical activity per week. Previous studies have supported the reliability and validity (against the accelerometer TriTrac-R3D) of the 7-Day PAR as an accurate measure for self-reported physical activity (Hayden-Wade et al., 2003). Further to the self-report measure of physical activity we objectively measured attendance to the ‘Active Lifestyle’ classes over the 10 week intervention using an electronic register.

### Data analysis

We carried out a paired samples *t* test to examine differences in need support conveyed via the messages in the pilot of the messages. We then used an independent samples *t* tests to test demographic, anthropometric and baseline differences (age, BMI, physical activity levels), between the intervention and control group at baseline. Further we used a MANOVA to test baseline differences between groups of motivational regulations. We employed intention to treat principles (last observation carried forward for those with missing data) to all analyses to minimise bias in the interpretation of the results. We removed one extreme outlier as we deemed the data reported for physical activity behaviour unrealistic. The individual case reported engaging in 450 min of moderate physical activity and 780 min of vigorous physical activity in a 1 week period.

Relationships between all outcome variables were examined at each time point using Pearson’s correlation. As the correlations among psychological need satisfaction variables, and behavioural regulation variable were moderate-to-strong (.21–.69), apart from introjected regulation (.08, −.4 and .13), the variables were included in separate mixed design multivariate analyses of covariance (MANCOVA) using SPSS (20.0) to determine the effect of group, time and their interaction on dependent variables; psychological need satisfaction (autonomy, competence and relatedness), behavioural regulations (amotivation, external regulation, introjected regulation, identified regulation and intrinsic motivation) separately. If the multivariate tests revealed a significant result, we conducted a follow up mixed design analysis (ANCOVA), using Bonferroni adjustment, to see where the differences occurred.

We assessed perceived autonomy support of the text messages and physical activity behaviour using separate mixed design analysis of covariance (ANCOVA). We treated individual perceptions of autonomy support from the class instructor as a time varying covariate (measured at mid and post intervention) to analyse the effects of the text messages whilst controlling for the effects of perceived autonomy support from the instructor in all variables. Tests of simple effects were conducted to probe the interactions further. Autonomy support was analysed using a 2 (condition; experimental vs. control) × 2 (time: mid vs. post) ANCOVA and physical activity was measured over 3 time points therefore a 2 × 3 mixed design ANCOVA was used for this variable. Effect sizes (partial eta squared) were classified based on Cohen’s cut-points (small = .01, medium = .06, large = .14) (Cohen, 1988). We used an independent *t* test to test group differences in class attendance.

## Results

### Pilot to test the text messages

Five individuals with expertise in SDT rated the randomised need supportive and neutral SMS according to their perceptions of need support from the messages. A paired sample *t* test showed that the two groups differed in terms of need support *t*(4) = 14.26, *p* < .01. Need supportive text messages (*M* = 6.01, *SD* = .45) were rated significantly higher on levels of perceived need support than the neutral text messages (*M* = 2.5, *SD* = .18). Small modifications were made to 8 of the messages following the feedback of the field experts. For example, on recommendation a change to the wording of a text was made to optimise supporting the need for autonomy. The message “It is understandable that you are concerned about your health. Joining an exercise class *is a* great step to leading a healthier lifestyle” was changed to “It is understandable that you are concerned about your health. Joining an exercise class *could be* a great step to leading a healthier lifestyle”.

### Descriptive data

Table 3 details descriptive statistics (means, standard deviation) for all variables and pin-points differences between groups and over time. We removed one item from autonomy need satisfaction from the text messages to ensure that median internal consistency coefficients were all .70 or greater and therefore demonstrating acceptable reliability according to DeVellis (2003).

**Table 3:** Descriptive statistics for perceived autonomy support, need satisfaction, motivation, and physical activity behaviour for both the intervention (SDT) and control group (C)

Variables	Pre		Mid		Post		4 Month	
	SDT	C	SDT	C	SDT	C	SDT	C
Perceived autonomy support								
Instructor/class	–	–	4.9 ± 1.0	4.83 ± 1.1	4.73 ± 1.05	4.46 ± 1.22	–	–
Text messages	–	–	4.13 ± 1.21	2.94 ± 1.29	4.43 ± 1.29	2.83 ± 1.2	–	–
*Perceived need satisfaction*								
Text messages								
Autonomy	–	–	4.33 ± 1.59	3.22 ± 1.4	4.82 ± 1.5	2.98 ± 1.33	–	–
Competence	–	–	4.43 ± 1.6	3.102 ± 1.59	4.69 ± 1.5	2.95 ± 1.61	–	–
Relatedness	–	–	5.31 ± 1.1	3.48 ± 1.33	5.3 ± 1.26	3.4 ± 1.19	–	–
Motivational regulations								
Amotivation	.24 ± .59	.31 ± .5	–	–	.27 ± .44	.39 ± .54	–	–
External	.40 ± .67	.58 ± .64	–	–	.43 ± .56	.69 ± .70	–	–
Introjected	1.22 ± .74	1.46 ± .96	–	–	1.33 ± .69	1.51 ± .96	–	–
Identified	3.06 ± .72	2.78 ± .85	–	–	3.08 ± .66	2.94 ± .64	–	–
Intrinsic	3.08 ± .68	2.64 ± 1.24	–	–	3.20 ± .61	2.88 ± 1.0	–	–
Physical activity (min)								
Moderate	70.48 ± 50.2	84.7 ± 79.78	–	–	97.74 ± 80.2	129.2 ± 184.07	125.8 ± 112.1	72.9 ± 80.9
Vigorous	15.8 ± 25.11	16.32 ± 27.6	–	–	71.77 ± 78.24	74.7 ± 83.08	85.58 ± 95.14	85.58 ± 86.1

*SDT* intervention group, *C* control group

### Randomization check

At baseline, we found no significant differences in demographic characteristics and baseline scores between the groups in age *t*(63) = .219, *p* = .83, BMI *t*(63) = −.82, *p* = .42, moderate intensity physical activity *t*(63) = −.85, *p* = .39, and vigorous intensity physical activity *t*(63) = −.07, *p* = .94. No group difference were seen at baseline for motivational regulations [F(5,59) = 1.21, *p* = .32, partial η^2^ = .01].

### Testing hypothesis 1: manipulation check of perceptions of autonomy support provided via the text messages

For a detailed presentation of all main effects and interactions of time and group see Table 4.

**Table 4:** Experimental main effects and interactions on autonomy support, psychological need satisfaction, motivational regulation, and physical activity behaviour

Dependant measure	Effect	*F*	*p*	Partial *η* ^2^
Autonomy support	T	(1,63) = .26	.47	.01
	G	(1,58) = 25.67	.01	.26
	T × G	(1,58) = 2.74	.10	.04
Autonomy	T	(1,62) = 2.29	.14	.01
	G	(1,61) = 20.5	.01	.19
	T × G	(1,58) = 5.92	.01	.12
Competence	T	(1,62) = .92	.34	.00
	G	(1,60) = 17.13	.01	.17
	T × G	(1,58) = .97	.33	.01
Relatedness	T	(1,60) = .03	.86	.01
	G	(1,59) = 38.95	.01	.28
	T × G	(1,56) = .55	.47	.01
Intrinsic motivation	T	(1,61) = 6.85	.01	.08
	G	(1,59) = 1.53	.22	.03
	T × G	(1,56) = .51	.48	.01
Moderate physical activity	T	(1,61) = 3.75	.06	.12
	G	(1,58) = .76	.39	.02
	T × G	(1,58) = 4.41	.04	.09
Vigorous physical activity	T	(1,60) = 38.13	.01	.36
	G	(1,58) = .48	.83	.00
	T × G	(1,58) = .00	.98	.00

T, main effect of time; G, main effect of time; T × G , time × group interaction

A repeated measures ANCOVA yielded a significant main effect for group [F(1,58) = 25.67, *p* < .01, partial η^2^ = .26] indicating that the intervention group did perceive greater levels of autonomy support from the text messages compared to the control group. No main effect was seen for time and no interaction effect was evident.

### Testing hypothesis 2: need supportive text messages and satisfaction of the three basic psychological needs

The initial 2 × 2 × 3 MANCOVA for basic psychological needs displayed a main effect for group [F(1,63) = 32.82, *p* < .05, partial η^2^ = .34] but not for time. Interaction effects were seen between time and group [F(1,63) = 4.44, *p* < .05, partial η^2^ = .07] and between need satisfaction and group [F(2,63) = 10.13, *p* < .01, partial η^2^ = .24]. Using a Bonferroni adjustment (*p* < .02), further mixed design ANCOVA showed significant differences, between the intervention and the control group, in the degree to which the needs for autonomy [F(1,61) = 20.5, *p* < .01, partial η^2^ = .19], competence [F(1,60) = 17.13, *p* < .01, partial η^2^ = .17] and relatedness [F(1,59) = 38.95, *p* < .01, partial η^2^ = .28] were fulfilled, with the intervention group significantly higher. Findings showed a main effect for group [F(1,62) = 20.5, *p* < .01, partial η^2^ = .19]. No main effect was seen for time. Findings revealed a significant interaction between time and group in the analysis of autonomy need satisfaction [F(1,58) = 5.92, *p* < .01, partial η^2^ = .12]. Further investigation showed that the intervention group reported increases in perceptions of autonomy need satisfaction from mid to post intervention (*p* < .05).^1^

### Testing hypothesis 3: need supportive text messages and behavioural regulations

The initial 2 × 2 × 4 MANCOVA for behavioural regulations revealed no significant main effects for group. However main effects were apparent for time [F(1,63) = 4.402, *p* < .05, partial η^2^ = .07] and motivational regulations [F(4,60) = 117.97, *p* < .01, partial η^2^ = .89]. Using Bonferroni adjustment (*p* < .01) further mixed design ANCOVA revealed no main effect for group although did for time in the analysis of intrinsic motivation [F(1,61) = 5.92, *p* < .01, partial η^2^ = .08]. This indicates that both groups increased their levels of intrinsic motivation over the course of the intervention. No main effects or interactions were evident for any of the remaining regulations.

### Testing hypothesis 4: the effects of need supportive text messages on physical activity behaviour (H4)

Analysis of moderate intensity physical activity yielded no significant main effect for group or time. However, there was a trend towards a difference of the main effect for time [F(1,61) = 3.75, *p* < .06, partial η^2^ = .12]. A significant group by time interaction effect was seen from baseline to the 4 month follow up [F(1,58) = 4.41, *p* < .04, partial η^2^ = .09]. Simple effects showed that the intervention group significantly increased their moderate intensity physical activity from baseline to the 4 month follow up (*p* < .05). Although the control group experienced an initial increase during the intervention (*p* < .05), their levels of moderate intensity physical activity had returned to baseline levels at the 4 month follow up. At the 4 month follow up the intervention group engaged in significantly more (*p* < .05) moderate intensity physical activity than the control group.

No group effects or interactions were evident (*p* > .05) in the analysis of vigorous intensity physical activity. A significant time effect was seen from pre-intervention to the 4 month follow up [F(1,58) = 38.13, *p* < .01, partial η^2^ = .36].

Analysis of the objective measure of class attendance showed no significant difference between the intervention (*M* = 12.52) and the control (*M* = 13) group (*t*(48) = −1.97, *p* = .84).

## Discussion

Existing research supports the use of text messages as a useful tool, in conjunction with other components, to encourage behaviour change within a variety of health contexts (i.e., smoking cessation, diabetes management, and sexual health). This is attributed to SMS based interventions being resource efficient (Haug et al., 2012); having the ability to reach a large proportion of the population instantly, and in a cost effective manner. Using a randomised controlled design our overarching aim was to investigate the effects of text messages, based on principles of SDT, compared to neutral text messages, in the promotion of physical activity behaviour and psychological well-being within a physically inactive population beginning a series of exercise classes.

SDT has successfully been applied to an exercise context (Teixeira et al., 2012) to explain how it is possible to optimise the quality of motivation by facilitating a need supportive environment. Traditionally, this need supportive environment has been enabled by a significant other. Our results indicate that participants in the intervention group perceived greater levels of autonomy support from the text messages compared to those in the control group, supporting that it is possible to facilitate feelings of autonomy support via a different social agent. It is known, through existing research, that perceived autonomy support can predict need satisfaction (Williams et al., 2006) and consequently, if needs are satisfied, autonomous motivation is optimised (Teixeira et al., 2012). The intervention group, compared to the control group, perceived significantly higher need satisfaction from the text messages for all three needs (autonomy, competence and relatedness) at both mid-point and at the post intervention measure. This therefore supports our second hypothesis, and tenets of SDT, that a need supportive text message based intervention can lead to increased need satisfaction and thus can be an effective tool used in the promotion of physical activity.

Satisfaction of the need for autonomy was the only psychological need to increase from mid to post intervention within the experimental group. This supports the notion that autonomy need satisfaction is more likely to occur, or continue to increase, in the latter stages of an intervention (Deci & Ryan, 2000) while competence and relatedness are likely to increase within the adoption phase of physical activity (Edmunds et al., 2008). However, at week 5, levels of need satisfaction in the experimental group was significantly higher than the control group which suggests that increases in feelings of autonomy also occurred early on in the intervention and continued to increase in the latter part of the intervention. It is therefore important to be mindful, when delivering an intervention aimed at increasing physical activity levels that although progressive focus can be made to increase autonomy need satisfaction, autonomy as well as competence and relatedness, should be targeted throughout the intervention. Our results extend the findings of Moustaka et al. (2012) and Edmunds et al. (2008) by showing that text messages can increase need satisfaction whilst controlling for the perceptions of autonomy support of the exercise instructor. Our findings also support those of Kinnafick et al. (2014) showing that need support can be achieved via unique contribution from different sources of support. Future research could investigate unique contributions of other sources of support (e.g., family member) beyond the intervention and the effect of text messages of those not starting a series of exercise classes.

We also hypothesised that those who received need supportive text messages would experience greater increases in self-determined motivation. We found no group differences for any behavioural regulations and therefore the third hypothesis is not supported. Intrinsic motivation did however, increase over time in both groups. Research has suggested that, in novice exercisers, intrinsic and identified behavioural regulations are likely to increase within the first 8 weeks whereas changes to more controlled regulations may take longer and are not stabilised until after 6 months of regular exercise (Rodgers et al., 2010). It is important, however, to note that the lack of significant findings could also be due to a ceiling effect of the instructor autonomy support (Fortier et al., 2012) and to the fact that the volunteers for this study were somewhat initially motivated to starting the exercise classes by attending a class within the existing programme in the first instance. Future research could examine this relationship further to ascertain whether similar results would occur in a group who were not initially motivated to begin a series of exercise classes. There was only one option with regards to buying credits to the classes (i.e., each individual would buy 1 batch of 10 credits and buy a further batch once they had been used up). Therefore we were not able to include the amount of credits bought as a covariate for initial levels of motivation in the analysis. Additionally, we were not able to control for different exercise elements within each class (e.g., toning, strength and aerobic elements) due to some classes containing a variety of elements (e.g., circuits).

Moderate intensity physical activity increased in the intervention group following the end of the intervention which supports our fourth hypothesis. SDT theorists posit that the satisfaction of the need for autonomy will result in positive behavioural outcomes (i.e., physical activity; Ng et al., 2012). The classes involved in the study were predominantly at a vigorous intensity suggesting that participants in both groups also engaged in moderate physical activity outside of the classes and those in the intervention group continued to do so after the end of the intervention. Attendance to classes did not differ between the groups which further suggest that the physical activity in the intervention group was undertaken outside of the class environment. It would be important, in future work, to examine the potential for autonomy supportive messages to promote physical activity both in and outside of exercise classes.

### Strengths and limitations

Using a physically inactive sample is a strength of our study given the apparent public health implications of increasing physical activity participation among this segment of the population. However, the sample size is relatively small and the participants all attended the university as students or staff and were predominantly young (*M* = 25.76 years *SD* = 10.2) and female (94 %). Although the size of the sample was small, the significant values detailed in Table 4 had partial eta square value classified as medium or large according to Cohen’s cut-points (Cohen, 1988), indicating medium to large effect size. In the future, researchers could carry out larger scale studies and include more diverse population groups to examine the generalisability of the findings.

In order to further improve the effectiveness of the messages to the individual, it would be useful if messages could have been sent at times that corresponded to when the participants were due to exercise. In order to keep in line with principles of need support, participants were not directed to attend specific classes but could make the choice themselves. This made it difficult to schedule messages according to when individuals intended to exercise. Further, by not prescribing specific classes it is possible, and it is important to be mindful, that participants of both the intervention group and control group may have attended the same classes. Existing research has varied greatly in their approach to the frequency of messages sent and suggests the inclusion of more detail on process of message development. We have included information regarding development and delivery of the messages in this manuscript. However, more research is also needed to examine the optimal frequency of text messages to affect both behaviour change and well-being outcomes.

Including a 4 month follow up and using validated measures are strengths of our study; however, using an objective measure of physical activity (e.g., accelerometers, such as the GT3X) could improve accuracy of physical activity measurement and capture an objective measure of lifestyle physical activity outside of the classes. We measured motives to exercise using the EMI-2 (Markland & Hardy, 1993) in order to individualise the text messages within the intervention group. Using the EMI-2 poses a potential limitation as the 14 categories of the questionnaire are made up of both reasons to exercise (e.g., enjoyment) and aims, or goals (e.g., weight management). Although the overarching aim of the messages was to focus on the personal value of both exercise motivation and exercise goals, these terms have previously been investigated as separate concepts (e.g., Vansteenkiste et al., 2004; Sebire et al., 2011). In order to isolate these separate concepts, future research should aim to distinguish between the underlying goal content, the reasons and the quality of motivation to pursue the goal. It is important to acknowledge that the effects of the text messages may be partly driven by the personalisation of the messages rather than solely need supportive communication. Therefore, future investigations could include an assessment of the individual’s motivational orientation and attempt to further disentangle the potential interactions between type of need-supportive messages, individuals motivational orientations and personalisation of the messages.

There are some limitations which should be considered when using mobile phones in behaviour change interventions. We only included participants in this study if they were already familiar with the use of mobile phone technology, and those challenged with illiteracy were excluded. Similar to previous text message based studies (Kim & Glanz, 2013), it was possible for us to view the delivery status of the text messages however we were not able to ascertain whether the texts had been opened or read. Similar research in the future could consider implementing a recall test to assess whether individuals had read and understood the messages. The inclusion of such a test could provide further insight into the reasons for the effect of the intervention. Further, although our results show that a text from a different social agent (mobile phone) can increase perceptions of autonomy support, it would be interesting to investigate the individual’s perceptions of the source of the text (e.g., human source or computer generated) and how that relates to perceptions of the social contextual environment.

Finally, due to the constraints of the university term, the study was only 10 weeks long and the recruitment period was restricted to 1 week and follow up period was 4 months following the end of the intervention. Future research would benefit from observing changes over a longer period of time and during all the seasons throughout the year.

## Conclusion

In conclusion, we reveal some evidence to support the use of need supportive text messages, although long term effects are less conclusive. Our findings suggest that it is possible for an individual (specifically young, healthy, and inactive females) to perceive support via an agency driven object, (i.e., text message) and thereby satisfy the three basic psychological needs as proposed by SDT. Whereas a need supportive text message is unlikely to replace traditional behaviour change interventions incorporating principles of SDT (Fortier et al., 2012; Ng et al., 2012), they may be an effective and a useful addition. Given the promising results of this study, and with further research, text messages grounded in SDT principles may improve existing interventions aimed at increasing quality of motivation and levels of physical activity in those who are insufficiently active.

## Electronic supplementary material

Below is the link to the electronic supplementary material.

Supplementary material 1 (DOCX 16 kb)

## Footnotes

1 Using Sobels (1982)’s mediation test, we examined the degree to which autonomy need satisfaction mediated the effects of autonomy support (via the text messages) on autonomous motivation in order to test the process model proposed by SDT (e.g., Edmunds et al., 2008; Teixeira et al., 2012). Although there was no direct effect of autonomy support of the text messages on autonomous motivation (β = .22, *p* *=* .08), Sobels test indicated that the mediation was significant (*z* = 2.29, *p* < .05) and thus supports the process model proposed by SDT.
