# Supplementary material for: The effect of need supportive text messages on motivation and physical activity behaviour
Source: J Behav Med. 2016 Feb 26;39:574–86. doi: 10.1007/s10865-016-9722-1 (PMC4942483; doi:10.1007/s10865-016-9722-1)
Supplement: Supplementary file 1 — Supplementary material 1 (DOCX 16 kb) [file 10865_2016_9722_MOESM1_ESM.docx]

Supplementary data: Pearson Correlations for Basic Need Satisfaction, Motivational Regulations and Physical Activity Behaviour at pre, mid, post intervention and at 4 month follow up

Variables Autonomy Competence Relatedness Amotivation External Introjected Identified Intrinsic Moderate Vigorous

SDT/C SDT/C SDT/C SDT/C SDT/C SDT/C SDT/C SDT/C SDT/C SDT/C

Pre

Motivational Regulations

Amotivation

External .61**/ .25

Introjected .30 / .22 .25 / .48**

Identified -.26 / -.31 -.34 / .23 .36* / .52**

Intrinsic -.21 / -.29 -.41 / -.25 .08 / .19 .69** / .61**

Physical Activity

Moderate -.16 / .04 -.18 / -.11 -.13 / .19 .25 / .21 .23 / .38*

Vigorous .09 / -.22 .02 / -.13 -.14 / 0.6 -.061 / .35* .09 / .23 -.34 / -.22

Mid

Basic Psychological Needs

Autonomy

Competence .52** / .69**

Relatedness .54** / .59** .45* / .68**

Post Intervention

Basic Psychological Needs

Autonomy

Competence .43* / .62**

Relatedness .65** / .59** .51** / .64**

Motivational Regulations

Amotivation .06 / -.02 .21 / .19 -.04 / .11

External -.23 / .12 .02 / -.13 -.01 / .10 .66**/ .27

Introjected -.17 / .27 -.19 / .02 -.23 / -.09 .23 / .26 .34 / .37*

Identified .31 / .23 .01 / .02 .08 / .03 -.27 / -.65** -.24 / -.27 .21 / .34*

Intrinsic .12 / .20 -.09 / .04 .05 / .11 -.45* / -.41* -.27 / -.29 -.13 / -.04 .65**/ .54**

Physical Activity

Moderate` .34 / -.01 .11 / -.33 .17 /-.11 -.08 / -.12 -.07 /-.08 -.05 / -.20 .13 / -.05 .09 / .07

Vigorous -.33 / .10 -.43* / .03 -.51**/-.06 -.28 / -.27 -.21 /-.04 -.03 / .01 .14 / .35* .24 / .29 -.29 / .43*

4 month

Physical Activity

Moderate

Vigorous -.39* / -.19

*Note:* * = *p* < 0.05; ** = *p* < 0.01; SDT = Intervention group; C = control group
